# Intersectionality Impacts Survivorship: Identity-Informed Recommendations to Improve the Quality of Life of African American Breast Cancer Survivors in Health Promotion Programming

**DOI:** 10.3390/ijerph191912807

**Published:** 2022-10-06

**Authors:** Rose Hennessy Garza, Michelle Y. Williams, Shana O. Ntiri, Michelle DeCoux Hampton, Alice F. Yan

**Affiliations:** 1 Joseph J Zilber School of Public Health, University of Wisconsin-Milwaukee, Milwaukee, WI 53205, USA; 2 Division of Research, Patient Care Services, Stanford Healthcare, Palo Alto, CA 94304, USA; 3 Division of Primary Care and Population Health, Department of Medicine, Stanford University School of Medicine, Palo Alto, CA 94304, USA; 4 Department of Family & Community Medicine, University of Maryland School of Medicine, Baltimore, MD 21201, USA

**Keywords:** African American women, breast cancer survivors, intersectionality, Black Feminist Thought, quality of life

## Abstract

(1) Background: African American women breast cancer survivors face unique experiences that impact their quality of life as they transition beyond treatments. Experiences may be complicated by living at the intersection of systemically oppressed identities, including gender, race, social class, and cancer-related disability. Using the Black Feminist Thought (BFT) framework and the PEN-3 cultural model, this qualitative study sought to: (a) understand African American women breast cancer survivors’ lived experiences; (b) examine how the multiple intersecting factors of race, gender, social class/socioeconomic status, and cancer-related disability impact their quality of life; and (c) inform future health promotion programming that is culturally relevant to AAWBCS to improve their quality of life. (2) Methods: Seven focus groups were conducted with 30 African American breast cancer survivors in a Midwestern metropolitan region. Focus groups were audiotaped and transcribed verbatim. Framework analyses were conducted to identify themes with NVivo qualitative analysis software. (3) Results: Four themes emerged: (a) caregiving roles provide both support and challenges for survivors, (b) the “strong Black woman” is inherent in survivor experiences, (c) intersectionality impacts survivorship, and (d) African American women resist oppression through culturally specific supports and advocacy. (4) Conclusions: The intervention point of entry should be at the peer support group level and centered on family and provide community-based support and services. Future research should move upstream to address social determinants of health, including racism, sexism, and ableism; there is a critical need to discuss how structural racism affects health care and develop interventions to address racial discrimination and racial bias in health care.

## 1. Introduction

Breast cancer is the most common cancer diagnosed in women and the second leading cause of cancer-related death among U.S. women. African American women experience disparities in breast cancer survival and are 42% more likely to die from breast cancer than their White counterparts [1]. While all breast cancer survivors face challenges, African American women encounter unique challenges. Compared to White survivors, African American women breast cancer survivors (AAWBCS) report a significantly worse health-related quality of life [2], and they are more likely to report economic and discriminatory barriers to seeking care and concerns related to caregiving roles. With more African American women surviving breast cancer, addressing their unique challenges, and building upon culturally specific strengths in health promotion programming will help improve their quality of life and survival [3].

To better understand the unique experiences and challenges of AAWBCS, this study applied the Black Feminist Thought (BFT) critical social theory as the framework. Established by Patricia Collins as a critical social theory, BFT centers the experiences of Black women while asserting that they experience life at multiple intersections of oppression, including race, class, gender, and sexual oppression [4,5]. This perspective recognizes that African American women, as a group, experience a different world than those who are not African American and women, and it highlights the importance of their unique culture [4,5]. BFT contains five core concepts [6]: (1) there is inherent value in African American women’s experiences; (2) African American women have a history of struggle against oppression; (3) African American women live at the intersection of race, class, and gender oppression; (4) African American women resist oppression through activism within their community; and (5) sexual politics and stereotypes have an impact on African American women’s lived experiences.

BFT has been used to explain the adverse health outcomes, including the lower rates of breastfeeding among Black women [7] and the higher rates of substance-use disorder recovery [8], HIV and sexual risk behaviors, and violence against Black women [9,10]. To our knowledge, only one study to date has applied the paradigm of BFT to assess AAWBCS’ unique stress as a result of living at these intersections of oppression [11]. Birthed out of the Black Feminist Movement, intersectionality “provides a framework to conceptualize how power relationships surrounding socially constructed roles of race, gender, and class merge to form oppressive social locations for women of color” [12,13]. Acknowledging intersecting identities is critical for understanding the unique experiences and challenges of AAWBCS, and this study will expand our understanding of African American women’s experiences with breast cancer.

**Figure 1:**
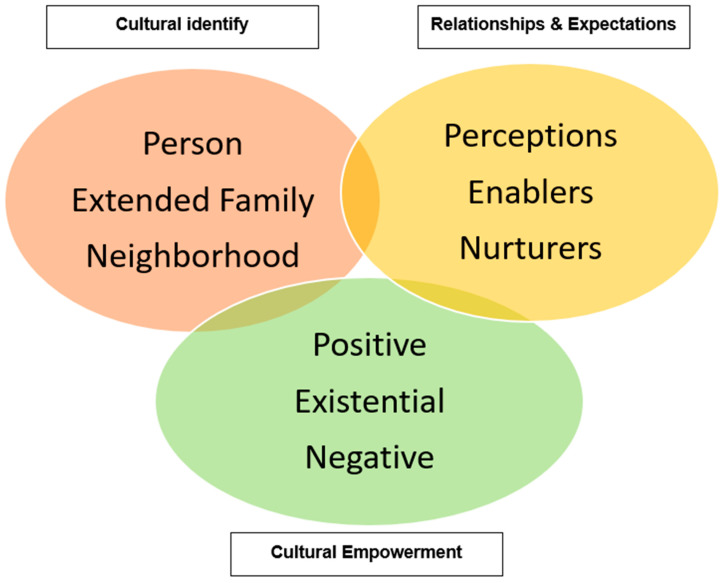
The PEN-3 cultural model.

Garnering “intersected” knowledge is not enough. Our ultimate goal is to reduce breast cancer disparities by translating this knowledge into health promotion programming. To inform the development of culturally relevant intervention programming, this study applied the PEN-3 cultural model [14,15]. The PEN-3 cultural model, developed by Dr. Collins Airhihenbuwa, focuses on placing culture at the forefront of health promotion [16]. The model consists of three dimensions of health beliefs and behaviors that are interdependent and interrelated. These dimensions include (1) cultural identity, (2) relationships and expectations, and (3) cultural empowerment. Within each dimension, three categories form the acronym PEN: Person, Extended family, and Neighborhood (cultural identity domain); Perceptions, Enablers, and Nurturers (relationship and expectation domain); and Positive, Existential, and Negative (cultural empowerment domain) [14,15]. According to the PEN-3 model, sustainable interventions for health behavior change must be “culturally anchored”; that is, built from (a) knowledge about the cultural context of health for a particular population and (b) appreciation of its community’s assets and challenges [14,15] (Figure 1).

Previous studies have used the PEN-3 model to inform various breast cancer research among African American women. For example, the PEN-3 model was used to guide qualitative studies to make cultural adaptations to breast cancer education pamphlets to (1) acknowledge African American cultural values related to community, self-reliance, and spirituality [17]; (2) optimize community-based and culturally sensitive recruitment strategies in epidemiology studies [18]; (3) identify perceptions, enablers, and nurturers of regular mammography [19]; and (4) inform the decision-support intervention messages for African American women newly diagnosed with breast cancer who are eligible for adjuvant therapy [20].

Guided by the BFT intersectionality framework and the PEN-3 model, this study will determine the intervention points of entry. These may occur at the level of persons (e.g., AAWBCS or health-care workers), extended family members (e.g., grandchildren, spouses/partners), or neighborhoods (communities). The second dimension, relationships and expectations, is used to determine the perceptions, enablers, and nurturers that impact survivors’ health behaviors, such as self-care or healthful lifestyle behaviors. Perceptions include the knowledge, attitude, values, and beliefs that either facilitate or hinder a woman’s behavior, e.g., reliance on God for cancer coping [21,22,23]. Enablers are community or structural factors (e.g., health-care resources, accessibility) that facilitate behaviors. Nurturers are reinforcing factors, such as the influence of family and kin from one’s social network. The third dimension, cultural empowerment, assesses the cultural appropriateness of health beliefs from the second dimension and categorizes these as “positive”, “existential” (neither positive nor negative), or “negative” (barriers).

To our knowledge, this study will be the first to explore the intersectional experiences of AAWBCS through a qualitative inquiry process. By centering intersecting identities, recommendations attend to AAWBCS in their entirety and avoid segmenting African American women’s identities. Using qualitative methods and applying the BFT framework and the PEN-3 cultural model, the current study’s objectives were three-fold: (1) to understand AAWBCS’ lived experiences, including their challenges and resilience during and following treatment; (2) to examine how the multiple intersecting factors of race, gender, social class/socioeconomic status, and cancer-related disability impact quality of life in AAWBCS; and (3) to inform future health promotion programming that is culturally relevant to AAWBCS to improve their quality of life.

## 2. Materials and Methods

### 2.1. Recruitment and Sample

Participants were recruited from health-care provider referrals, African American breast cancer support groups, and community agency referrals in a Midwest metropolitan area of the United States. In addition, recruitment flyers were placed in various community locations (i.e., local churches, hair salons, civic groups, and nonprofits). The inclusion criteria were: (1) self-identifying as a Black/African American woman and currently living in the study area; (2) adults aged ≥18 years; (3) diagnosed with noninvasive (stage 0) or operable invasive breast carcinoma (stage I, II or III breast cancer diagnosis); (4) not diagnosed with any other types of cancer; (5) finished “active” cancer treatment (defined as surgery, chemotherapy, and/or radiation treatment) in at least a six-month span; (6) ability to read and speak English; and (7) be accessible by telephone. Exclusion criteria included being currently pregnant or undergoing therapy for life-threatening illnesses. Written informed consent was obtained at the beginning of the focus group sessions.

### 2.2. Data Collection

Recruitment, enrollment, and the focus groups occurred during 2016–2017. Focus groups were conducted in a small community-accessible conference room. Each group lasted approximately two hours and was audio recorded and moderated by a trained research assistant, who is an African American woman. The moderator followed a standardized group discussion guide. This study used a protocol starting with open-ended exploratory questions related to what it means to be an African American woman who survived breast cancer in general and within the African American community. The interview guide was developed in alignment with the BFT theory to reflect AAWBCS’ lived experiences at multiple intersections of oppression, including race, class, gender, and sexual oppression [4,5]. Then, participants discussed questions related to the impact of breast cancer and shared their experiences during and post-cancer treatment periods. Two co-facilitators summarized individual comments on a notepad. Participants were compensated with a $20 gift card. The study was approved by the Ethics Committee of the Institutional Review Boards (IRB) and verified to conform to the provisions of the Declaration of Helsinki.

### 2.3. Data Analysis

Audio recordings were transcribed verbatim and analyzed thematically. Qualitative analyses were implemented using computer-assisted coding and memos (NVivo 11, a software package for qualitative data management and analysis; QSR International). Qualitative data analysis procedures were based on the framework analysis methodology by Krueger and Casey [24]. The analysis was an iterative process that involved the constant comparison method developed by Glaser and Strauss [25,26,27,28]. Using an inductive approach, themes were added as they appeared in the transcripts to guard against selectivity. Inductively, themes were sorted in relation to the theories used to guide the analysis. Themes related to identity, particularly race, gender, social class, and disability, were coded to analyze the data through the lens of BFT [4,5], with an emphasis on positive, existential, and negative themes related to perceptions, enablers, and nurturing characteristics in accordance with the PEN-3 model [14,15]. This approach allowed us to combine the theories within our analysis to ensure that themes of the PEN-3 model were centered around the lives of African American women and based in BFT. While one researcher finalized codes for the manuscript, three research assistants met weekly for three months to discuss the themes and trends in the data coding until all focus groups had been reviewed. This process assured internal quality control and helped validate the themes and codes through independent coding and group consensus. Data collection and/or analysis continued until data saturation was reached; defined as no new themes having emerged from focus groups [29,30].

## 3. Results

### 3.1. Characteristics of Participants

A total of 30 survivors participated in seven focus groups, with an average of four participants per group. Of the 30 participants, 70% percent were 55 or older. Based on self-reported marital status, twelve (40%) had never been married. In regard to educational attainment, 23.3% of participants had completed some high school, 23% were high school graduates, 37% had completed some college or technical school, and 16.7% were college graduates. Regarding income, more than half of the participants (56.7%) reported an annual household income of less than $20,000.

### 3.2. Focus Group Findings

Four themes emerged in the analysis of data: (1) caregiving roles provide both support and challenges for survivors, (2) the “strong Black woman” is inherent in survivor experiences, (3) intersectionality impacts survivorship, (4) African American women resist oppression through culturally specific supports and advocacy. Incorporating these themes, the PEN-3 cultural model provides culturally responsive recommendations for health promotion programming.

#### 3.2.1. Theme 1. Caregiving Roles Provide Both Support and Challenges for Survivors

Traditional gender roles may position women to take on more caregiving in their families, which include cooking, cleaning, and caring for parents, children, and grandchildren. These roles emerged as both motivators for survivorship and challenges in maintaining health. Survivors expressed that caring for their families provided them with purpose, self-worth, and motivation. One participant stated:

*“Because I tell my grand babies I live for them. They is what gives me joy. They is what give me the strength I need because when I feel depressed, I feel lonely, I feel like I don’t want to go outside, I don’t want to make no phone call, I got to keep it real … And then when I get that phone call or they just pop up at my door, there it is. It’s like … something just click. You know what I’m saying? And my grandkids is what give me life. And I tell them that all the time, ‘I live for y’all. Y’all give me the strength, the joy that I need to keep keeping on.’”*

Another participant shared experiences caregiving for her grandson in the morning. She explained his concern for her slipping in the cold while they waited for his bus:

*“‘Go back, grandma. You gonna fall out and who gonna pick you up! … Go back, grandma … it’s slick out here,’ he can tell me, ‘You gonna fall and who gonna pick you up?’ I said, ‘You gonna get me up…’ But I- it’s- that keeps me going.”*

While family demands are motivating, they can take a toll and impact on survivors’ physical and mental health. Survivors report exhaustion and fatigue in caregiving and little external social support to assist in these gender-defined roles. One participant shared:

*“Yup. I did, because I put my foot down with my son and told him … ‘You got to come get your kids. I’ve gotta bathe them, I gotta feed them. I’m getting old, tired, whatever.’ And I’m like that, getting so I can’t go through this … I can’t cuz I get too tired. I be so tired … I said, ‘Y’all gonna send me to my grave early.’”*

For those in heterosexual relationships, women rarely mentioned men assisting in caregiving roles, or shared that men presumed women would maintain their caregiving roles through their cancer recovery. As one participant expressed, expectations fell on her shoulders:

*“My stuff is always, my husband’s, nothin’s never wrong. They always pass it all on to me. Um. I don’t know. I work every day. I take care of my mom. She’s 94. And I’m not, I’m busy doin’ whatever I need to do. Drive. Do normal. Men think we’re invincible, so, you know, do what you have to do.”*

#### 3.2.2. Theme 2. The Strong Black Woman Is Inherent in Survivor Experiences

Previous researchers have labeled the construct that African American women use to describe their lives and experiences as the “strong Black woman”, which is characterized by the promotion of unfaltering toughness (strength), resilience, self-containment, and self-sacrificing (denial of self-needs) [31,32]. The embodiment of strong Black woman reinforces historical stereotypes in the U.S., while also providing a positive identity for survival. In Allen et al.’s study of racial discrimination, there were protective health benefits of identifying with this superwoman schema but also aspects that worsened health risks, such as feeling obligated to help others [33]. Survivors identify as being strong and resilient African American women. As one participant shared:

*“Because even through all this I’m goin through I still manage to be there for my sister, my family. You know, I’m doin that. Because I’m strong enough to do it. Even when I was like really sick and from like all we need to be, I was out there tryin to do somethin’.”*

Survivors were able to draw upon a strength identity and continue many aspects of their lives, sometimes without their family knowing they had breast cancer. Some women shared that they did not disclose their diagnosis to anyone. One woman waited a year to tell her children she had breast cancer, stating:

*“I didn’t want to hurt them. And I didn’t tell anybody in the family … Because I was afraid I would devastate them … And I lived with it for that year and then when I did tell them, that devastated them more.”*

While adhering to the strong Black woman role can cause self-denial of needs, seeking help was necessary to deal with struggles in survivorship, including social anxiety, depression, phobias, and panic attacks. This was reflected by one survivor who stated:

*“I couldn’t come out my house for almost two years … after that … I was having really panic attacks and instead like I would just get really hot. It felt like, you know how you could be in a, uh, the lunchroom, how you just hear voices, just you hear everything everybody is saying…”*

In line with emerging research, themes suggested that African American breast cancer survivors identify with the strong Black woman role and that adherence to this role may be both protective and detrimental to various aspects of survival and quality of life.

#### 3.2.3. Theme 3. Intersectionality Impacts Survivorship

The third theme is how intersectionality impacts survivorship. It is closely related to the fourth concept of BFT—that African American women live at the intersection of race, social class/socioeconomic status, and gender oppression. The oppression that comes from intersecting and marginalized identities can make survivorship for African American women more challenging. Survivors shared concerns related to finances, unsafe neighborhoods, health insurance, and access to healthy food. Having a low income left some survivors dependent on financial assistance/grants, and while many shared positive experiences, these support mechanisms were difficult to navigate and frequently provided only short-term solutions. The limited and short-term nature of financial assistance left some women constantly needing to seek assistance. However, dealing with disability from cancer made navigating these processes more difficult. Exacerbating these challenges, a few participants shared experiences of racial discrimination as an additional barrier to receiving services. As one participant shared:

*“I don’t mind exposing his name because he deserved exposing, Dr. XX at the time, was at the XYZ hospital but wouldn’t come and see me. And um, so I got out of the hospital and I went to his office and I asked why didn’t you come and see me. He said “Well, there’s nothing we can do for you.” And my husband was like “Man, you her doctor, why wouldn’t you come and at least check in on your patient, and we actually saw you walking around the hall and you totally ignored her.”*

Another participant shared her painful experience because the doctor did not acknowledge or know who she was:

*‘’I had that experience with one of my oncologists when I went through my cancer thing and it really, really hurt my feelings just the fact that he had, didn’t quite acknowledge me, um, you know when I was in his office getting chemo. One day I was sitting for some test that he had referred me to and he walked right past me like he didn’t even know who I was. And you know, seeing his face every day for chemo. So when he did that to me and he didn’t acknowledge the face and then every time I came with him with questions, he just like brushed it off. He- it’s like my family would ask him things about me and what I was going through and he just- he wouldn’t give us answers. He just basically just brushed it off.”*

Social class impacts where women live, and the intersection between race and social class positions people of color in racially segregated neighborhoods with more deprivation and less access to health and social services [34]. Some women reported low levels of safety in their neighborhoods and a lack of local grocery stores that offer affordable, healthy food. As one participant shared:

*“I don’t know, if you want to do something you have to go so far out, stores have moved out of the neighborhoods … Because it used to be, maybe it still, can you go, you used to be able to go to market, general hospital or whatever, but they’ve moved. All of those things, out of your neighborhood. You don’t have access to it.”*

The challenge of seeking resources is exacerbated by living at the intersections of oppression. If African American women did not live in racially oppressed and isolated neighborhoods, their services “would not be so far out”, and traveling further would not be as challenging if they were not survivors dealing with health impacts from cancer.

#### 3.2.4. Theme 4. African American Women RESIST Oppression through Culturally Specific Support and Advocacy

African American women breast cancer survivors resist oppression through activism and advocacy within their community. Survivors mobilize in culturally relevant ways that allow them to thrive, particularly through spirituality and with their churches. They shared a desire to educate others about breast cancer, promote screening, and spread hope. Survivors participate in breast cancer walks, mentor others, and host their own events. As one participant shared:

*“Like my cousin, she throws a pink party every year, and it’s just for people in the family to come, and she has like brassieres and stuff like this just to inform them, educate, because, you know, back in the day it was something you didn’t really know about. You shied away. You thought it was something negative. But there’s more information now. And we have to be that support system. You don’t know who’s next? So you have to build each other up and inform. We’ve got to get this education.”*

This advocacy was further fueled by women’s attitudes and beliefs about survival, resiliency, and hope. This is embodied in the words of one participant:

*“You know, I’ma live or die, but so in the meanwhile, it’s full, the doors open for me to challenge whatever I face and my family faces and my community faces that I don’t think is right. So, it gave me a voice and that was the part about staying strong because in order to have a voice, you have to stay strong.”*

## 4. Discussion

This qualitative study explored AAWBCS’ lived experiences and examined how the multiple intersecting factors of race, gender, social class/socioeconomic status, and cancer-related disability impact their quality of life. The findings of this study were also used to inform future health promotion programming that is culturally relevant to AAWBCS to improve their quality of life. This study contributes to the field by collectively applying the BFT intersectionality framework and the PEN-3 model.

According to the BTF framework [6], the core concept essential to BFT is a legacy of struggle against oppression. Throughout the history of the United States, the interrelationship of white supremacy and patriarchy has characterized Black women’s reality as a situation of struggle [6]. Despite differences created by historical era, ethnicity, social class/socioeconomic status, and sexual orientation, the legacy of the struggle against racism and sexism has led to Black women’s independence and self-reliance, as well as served as a common thread that binds African American women together in the United States.

Among the few studies to use BFT as a paradigm for interpreting the unique experiences of AAWBCS, our findings are consistent with the recent study by Armour-Burton and colleagues [11], which indicated that African American women with breast cancer live at the intersection of race, social class/socioeconomic status, and gender oppression. The oppression that comes from having intersecting and marginalized identities can make survivorship for African American women more challenging. Racial health disparities occur when these socially constructed and oppressive identities come together with various manifestations of structural racism, including but not limited to differential pay, residential segregation, unequal distributions of resources (i.e., the lack of social services, affordable healthy food, and safe neighborhoods for exercise), economic opportunities, education, and a lack of access to health care in the community [34,35]. In addition, the racial discrimination and racial bias that AAWBCS experience negatively impact their quality of health care and health outcomes—“racial ethnic minorities receive lower-quality health care than white people regardless of income and insurance coverage. [36,37] ” The challenge of seeking resources is exacerbated by living at the intersections of oppresssion.

The BFT intersectionality framework also helps explain the theme “strong Black woman” as it allows us to center AAWBCS’ challenges as we consider the ways in which racism and gender-based oppression shape survivors’ mental health outcomes. Their race-gender identity affects African American women’s stress experience, coping responses, and mental health. Consistent with our findings, previous studies indicated that the perception and endorsement that African American women are naturally strong, resilient, self-contained, and self-sacrificing provided African American women with protection against the numerous stressors they encounter daily and was reported as protecting them from the adverse health impacts of racial discrimination [33]. However, qualitative evidence also suggests that the “strong Black woman” label positions survivors with long-term disabilities to deal with additional health-related challenges when adhering to expectations of “being strong, self-contained and self-sacrificing.” This situation consequently limits some African American women’s ability to cope healthily and can potentially exacerbate the negative mental health outcomes of stress [31]. The need to address emotional and mental health problems was also observed in our participants.

The PEN-3 cultural model provides recommendations for culturally relevant interventions. In applying the PEN-3 model, the findings provided “insightful” and culturally relevant recommendations that can be used to inform future health promotion programming specifically for AAWBCS. These recommendations reinforce strengths-based solutions. Based on focus group findings related to AAWBCS’ intersectionality of cultural identities, the intervention point of entry should be at the peer support group level and centered on family. Three primary recommendations are described that future health promotion programming with African American women breast cancer survivors should: (1) create space to discuss identity, strengths, and role adherence/stress; (2) center services around family and community context, acknowledging the unique roles of each family member (spouse/partners, sons and daughters, and grandchildren); and (3) acknowledge existing advocacy by survivors and promote these efforts.

Our findings suggested that the intervention point of entry should be at the peer support group level. Emerging evidence suggests that peer support is an effective strategy for AAWBCS. Those who received peer support reported greater access to and utilization of support resources, an increased ability to manage breast cancer-related stress, and an improved quality of life compared to survivors without peer support [38]. Data suggests a group-based, adapted version of the Stanford Chronic Disease Self-Management Program for Cancer Survivors can reach and appeal to survivors, provide peer support, and address common self-managing needs for survivors [39]. To address intersectionality, caregiving roles, and the powerful but taxing role of the strong Black woman, health promotion programming should create space for AAWBCS to discuss identity, strengths, role adherence, and the related psychological stress. Programming should encourage connection, and space should be provided to process gender- and race-based discrimination together. Research indicates that AAWBCS welcome and actively participate in support groups that are culturally appropriate [21].

The focus on group reflection may be particularly crucial, as previous research indicates that women transitioning from active breast cancer treatments want to connect with other survivors after treatment when there is less support from the medical community [40,41]. Flannery et al. [42] discuss the need for African American women breast cancer survivors (AAWBCS) to engage in social support “on [their] own terms”. Finally, most participants found balancing care for themselves, their family, and their community challenging. Since caregiving motivates survival, providing space for discussion may help survivors process these experiences, consider management strategies, and ideally normalize self-care with their peers.

In addition to creating space for peer support, we suggest interventions that consider a strengths-based and family-centered approach. Similar to previous research, our study revealed that some AAWBCS experience loving support from family while others lack family support [43]. Research on breast cancer survivors suggests that within a year of completing treatment, the quantity of social support provided to survivors significantly decreases and is associated with increased depression symptoms and stress [40]. Programming that builds bonds with family members will enhance existing sources of support. Some women shared that they had male partners/spouses who were great sources of support. Programs could collaborate with men who are partners of survivors to share their experiences supporting the women in their lives with breast cancer, as previous research suggests that men wish to support their wives who have breast cancer in “ways that are not familiar to them” [44]. Other research with AAWBCS suggests that family-centered programming should also welcome extended family or friends who serve in the role of caregivers [38,42].

In addition to building family bonds, health promotion programming may be improved by centering services around a community context. Program planners should consider and continue faith-based interventions [22]. Programming to decrease stress, lower anxiety, and improve mental health for AAWBCS may particularly benefit from religious/spiritual interventions (RSIs); a meta-analysis of RSIs found significant reductions in all three categories when intervening in already religious populations [45].

Our work reinforced a core tenant of BFT found in similar studies, which is that AAWBCS resist oppression through engagement in activism with their family, social network, and larger community [42]. In fact, the strong Black woman collective theory [46] suggests that African American women reappropriate the image of “being strong Black women” and “regulate strength in each other to promote solidarity within the group and confront external hostilities collectively.” Health promotion professionals can partner with survivors and support these efforts by celebrating with them, providing resources, and promoting event idea sharing. Breast cancer awareness events offer a public platform to highlight the experiences of AAWBCS. Since survivors want to change the association of cancer with death in the African American community, it is crucial that programming allows women to celebrate survivorship in a community context. Research supports the desire of AAWBCS to give back to their community, further supporting this intervention component [41]. On a structural level, health promotion professionals could partner in activism beyond awareness events. Since many barriers to survivorship, quality of life, and quality of health care are due to structural barriers based in social class, race, gender, and disability, professionals should collaborate with AAWBCS to address systematic barriers related to service access and utilization, bias and discrimination by health-care professionals, and social norms related to gender and race [36,37]. On a societal level, health promotion programming should aim to prevent racism, sexism, and ableism, fundamental causes of health that may exacerbate adverse health outcomes in AAWBCS.

### Limitations

First, this study reveals the unique experiences of the AAWBCS who participated in the study and may not reflect the larger population of AAWBCS in the recruitment region or beyond. For example, the sample was mainly at the lower socioeconomic level and were all U.S.-born African Americans. As a result, findings may not be generalizable to all AAWBCS. Specifically, there may be differences in the lived experiences of African American women from different socioeconomic groups or Black participants who might not be U.S.-born. Second, information about gender identity or sexual orientation and other contextual, demographic information was unavailable to use in further interpreting findings. Third, social desirability bias is also inherent in the study design, as participants may have been hesitant to share their actual experiences, beliefs, or opinions in a group setting.

## 5. Conclusions

This study is one of the few studies to use the BFT framework as a paradigm to interpret the unique experiences of AAWBCS. Guided by the BFT intersectionality framework and the PEN-3 model, our findings provide insights that can inform interventions for promoting the health and well-being of AAWBCS. The intervention point of entry should be at the peer support group level, center on family, and provide community-based support and services. Future research should move upstream to address social determinants of health [47], including racism, racial bias, sexism, and ableism—fundamental causes of health that may exacerbate adverse health outcomes in AAWBCS. It is also critical to discuss how structural racism affects health care and to develop interventions to address racial discrimination and racial bias in health care. While effective interventions can be more complex or resource-intensive when moving upstream than providing counseling on cancer-related stress alone, health-care organizations and policymakers hoping to reduce cancer health disparities and achieve health equity at the population level must commit the effort and investment required to achieve this goal.

## Data Availability

Not applicable.
